# Persistent Endothelial Activation and Inflammation After *Plasmodium falciparum* Infection in Malawian Children

**DOI:** 10.1093/infdis/jit419

**Published:** 2013-09-17

**Authors:** Christopher A. Moxon, Ngawina V. Chisala, Samuel C. Wassmer, Terrie E. Taylor, Karl B. Seydel, Malcolm E. Molyneux, Brian Faragher, Neil Kennedy, Cheng-Hock Toh, Alister G. Craig, Robert S. Heyderman

**Affiliations:** 1 Malawi-Liverpool-Wellcome Clinical Research Programme; 2 Blantyre Malaria Project; 3 Department of Paediatrics, Queen Elizabeth Hospital, University of Malawi College of Medicine, Blantyre; 4 Liverpool School of Tropical Medicine; 5 Institute of Infection and Global Health, Liverpool University, United Kingdom; 6 New York University School of Medicine, New York; 7 College of Osteopathic Medicine, Michigan State University, East Lansing

**Keywords:** *Plasmodium falciparum*, pathogen burden, endothelial activation, endothelial dysfunction, inflammation, cardiovascular disease, stroke, pediatric

## Abstract

Endothelial dysregulation is central to the pathogenesis of acute *Plasmodium falciparum* infection. It has been assumed that this dysregulation resolves rapidly after treatment, but this return to normality has been neither demonstrated nor quantified. We therefore measured a panel of plasma endothelial markers acutely and in convalescence in Malawian children with uncomplicated or cerebral malaria. Evidence of persistent endothelial activation and inflammation, indicated by increased plasma levels of soluble intracellular adhesion molecule 1, angiopoetin 2, and C-reactive protein, were observed at 1 month follow-up visits. These vascular changes may represent a previously unrecognized contributor to ongoing malaria-associated morbidity and mortality.

*Plasmodium falciparum* causes approximately 300 million clinical episodes of malaria every year, mainly in sub–Saharan Africa [1]. In hyperendemic regions, children may receive >1 infective bite per day and experience repeated clinical episodes [2]. Although acute infection is directly responsible for 750 000 deaths [3], it is increasingly apparent that there is an additional indirect burden of mortality. Several studies have shown that reducing malaria transmission in a population may be followed by all-cause childhood mortality reductions as high as 70%, a greater effect on mortality than can be attributed to reducing parasitemic febrile illness alone [2, 4, 5]. The cause of this additional burden remains uncertain. Given the alteration in vascular endothelial function during acute malaria pathogenesis, 1 plausible mechanism is that endothelial dysfunction persists beyond the acute febrile episode and influences the risk or outcome of reinfection or other diseases.

We postulated that malaria infection induces persistent alteration in endothelial activation. We tested this using a panel of plasma endothelial markers linked to key endothelial functions: soluble ICAM-1 (sICAM-1) and sE-selectin for endothelial activation; angiopoetin 2 (Ang2) for angiogenesis and endothelial quiescence; C-reactive protein (CRP) for inflammation; prothrombin fragment F1 + 2 for a procoagulant state; and soluble thrombomodulin (sTM) for endothelial damage.

## METHODS

Recruitment took place at the Queen Elizabeth Central Hospital, Blantyre, Malawi between January 2010 and June 2011.Children aged 1–12 years were recruited into 1 of 4 clinical categories: (1) cerebral malaria (CM) cases, who fulfilled World Health Organization (WHO) diagnostic criteria [6] and had at least 1 feature of malarial retinopathy [7]; (2) children with uncomplicated malaria (UM); (3) children with aparasitemic mild febrile illness (MF); and (4) aparasitemic healthy controls (HC), recruited from well children attending elective surgery. Children were included in the UM and MF groups if they had an axillary temperature >37.5°C and no features of organ compromise as indicated by WHO criteria [6]. Parasitemia was screened for on admission and in follow-up visits using a combined *P. falciparum* histidine rich protein and pan-malarial lactate dehydrogenase rapid diagnostic test kit, and in patients in whom both parameters were positive, parasitemia was confirmed on blood smear. In all patient groups, children were excluded if they had evidence of meningitis or severe malnutrition, had WHO stage 4 human immunodeficiency virus infection, or were on antiretroviral therapy. Informed consent was given by the parent or legal guardian of all children enrolled in the study. The study was approved by the College of Medicine Research Ethics Committee in Malawi (No. P.02/10/860) and by the Liverpool School of Tropical Medicine ethical board in the United Kingdom (No. 09.74).

CM patients were managed on a pediatric high-dependency unit according to established protocols. They were treated with intravenous quinine for a minimum of 3 doses, until either 2 consecutive negative malaria blood films or until they could tolerate oral medication. They were then given a 3-day course of oral lumefantrine-artemether according to national treatment guidelines. UM patients were treated with a 3-day course of oral lumefantrine-artemether, and MF patients were given treatment as deemed clinically appropriate by the local clinical team. Patients were excluded from further analysis if they had fever or were parasitemic on the day of follow-up.

Citrated plasma samples were taken at presentation and at 1 week and 1 month follow-up visits, and plasma was stored at −80°C until required. sICAM-1, sE-Selectin, sTM, CRP, and Ang2 concentrations were determined using commercial enzyme-linked immunosorbent assay kits and F1 + 2 using the Enzygnost F1 + 2 micro kit.

Statistical analysis was performed using Stata version 11 and Prism version 5.0 software. Skewed data were log-transformed, and differences between groups were compared by 1-way analysis of variance, with the Tukey honestly significant difference test to adjust for multiple comparisons. All tests were 2-tailed, and significance was set at the 5% level.

## RESULTS

Eighty-eight children with MF, 84 children with UM, 18 children with CM, and 36 HC children were recruited (see Table 1). Approximately 50% of these were followed-up to day 28 (Supplementary Figure 1).

**Table 1.** Clinical Characteristics and Soluble Plasma Markers in Children With Cerebral Malaria, Uncomplicated Malaria, and Mild Febrile Illness and in Healthy Controls

Characteristic	Healthy Controls (n = 36)	Mild Febrile Illness (n = 88)	Uncomplicated Malaria (n = 84)	Cerebral Malaria (n = 18)
Age, years, mean (95% CI^a^)	4.6 (3.7–5.7)	3.2 (2.8–3.6)*	4.7 (4.1–5.3)	4.1 (3.3–5.2)
Female sex, No. (%)	13 (36)	35 (40)	47 (56)	8 (44)
Clinical parameters on day 0, mean (95% CI)				
Axillary temperature	36.6 (36.5–36.7)	38.4 (38.2–38.5)***	38.5 (38.4–38.7)***	39.0 (38.5–39.6)***
Heart rate, beats/minutes	109 (104–114)	127 (119–135)***	130 (124–137)***	145 (129–163)***
Systolic blood pressure, mm Hg	107 (101–113)	114 (110–117)*	113 (110–117)*	97 (92–102)**
Respiratory rate, breaths/minutes	28 (26–30)	30 (29–31)	28 (27–29)	46 (42–51)***
Glucose, mmol/L	5.0 (4.8–5.2)	5.1 (4.9–5.3)	5.7 (5.4–6.0)**	5.6 (4.6–6.9)*
Lactate, mmol/L	1.8 (1.7–2.0)	1.7 (1.6–1.9)	2.3 (2.1–2.5)	5.2 (3.9–6.9)***
Hemoglobin, g/L	10.4 (9.9–11.0)	10.7 (10.3–11.1)	9.0 (8.6–9.4)**	6.1 (5.4–7.0)***
Platelets, ×10^9^/L	380 (347–416)	300 (267–338)*	110 (93–131)***	31 (21–46)***
Parasitemia, parasites ×10^3^/µL	0	0	20 (9.9–40)	68 (27–171)
HIV^b^ positive, No. (%)	0 (0)	4 (4.8)	3 (3.5)	2 (11.1)
sICAM-1, pg/mL day 0	198 (160–247)	316 (295–339)***	464 (295–505)***	624 (377–1033)***
sICAM-1, pg/mL day 7	(…)	258 (235–282)	343 (299–394)***	478 (364–626)***
sICAM-1 ,pg/mL day 28	(…)	269 (242–299)*	277 (251–304)**	291 (201–421)
sE-selectin, pg/mL day 0	68 (57–82)	122 (105–142)***	147 (130–160)***	205 (158–266)***
sE-selectin, pg/mL day 7	(…)	76 (66–87)	79 (68–91)	109 (77–154)*
sE-selectin, pg/mL day 28	(…)	79 (68–92)	82 (74–91)	93 (64–135)
Ang2, pg/mL day 0	232 (207–261)	382 (339–431)***	579 (523–641)***	1536 (1190–1982)***
Ang2, pg/mL day 7	(…)	383 (333–440)***	442 (389–502)***	548 (401–749)***
Ang2, pg/mL day 28	(…)	311 (275–350)**	320 (285–359)**	307 (213–443)
sTM, pg/mL day 0	4.7 (4.2–5.2)	4.5 (4.2–4.9)	6.0 (5.4–6.5)*	9.1 (7.5–11.1)***
sTM, pg/mL day 7	(…)	4.7 (4.3–5.1)	5.0 (4.4–5.6)	5.6 (4.3–7.2)
sTM, pg/mL day 28	(…)	5.2 (4.8–5.6)	4.6 (4.1–5.3)	4.1 (3.4–5.1)
CRP, mg/mL enrollment	0.41 (.23–.74)	26.7 (20–35)***	75 (61–92)***	149 (111–199)***
CRP, mg/mL day 7	(…)	3.0 (2.0–4.4)***	5.3 (4.2–6.7)***	16.0 (8.3–32)***
CRP, mg/mL day 28	(…)	0.62 (.37–1.0)	0.73 (.48–1.1)	12.7 (3.1–52)***
F1 + 2 , pg/mL day 0	176 (140–220)	142 (120–167)	219 (180–268)	376 (245–577)*
F1 + 2 , pg/mL day 7	(…)	176 (135–229)	220 (186–260)	500 (279–898)***
F1 + 2 , pg/mL day 28	(…)	174 (138–221)	142 (111–182)	180 (105–310)

For each variable, differences between healthy controls and other patient groups were examined using a Fisher's exact test (categorical variables) or 1-way analysis of variance (continuous variables) with the Tukey honestly significant difference test to adjust for multiple comparisons. Comparisons were not made for parasitemia or HIV. Abbreviations: Ang2, angiopoetin 2; CI, confidence interval; CRP, C-reactive protein; HIV, human immunodeficiency virus; sICAM-1, soluble intercellular adhesion molecule 1; sTM, soluble thrombomodulin. *P < .05, **P < .01, ***P < .001.

^a^ All mean values are geometric means and their 95% confidence intervals.

^b^ HIV was tested for using rapid testing.

**Figure 1.**
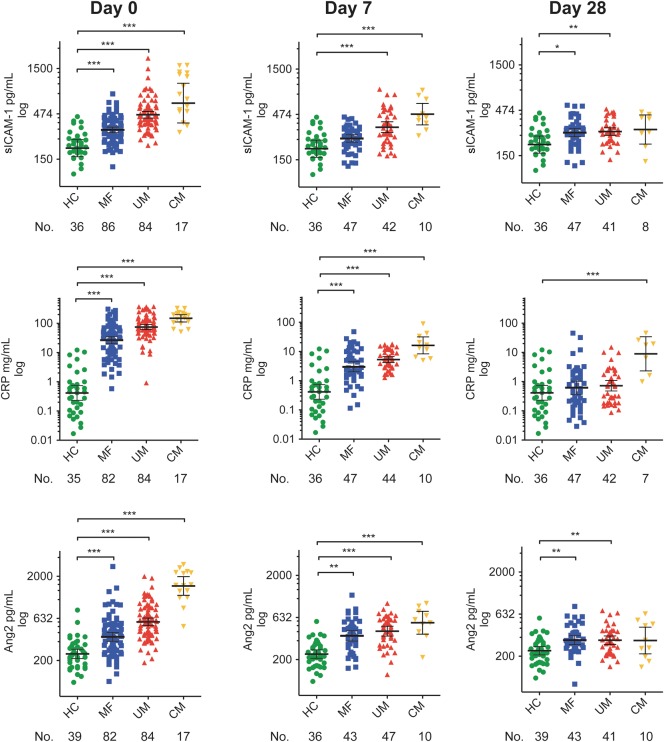
Plasma markers at admission and at follow-up in Malawian children with malaria, nonmalarial febrile illness, and cerebral malaria compared with healthy controls. Levels of soluble intercellular adhesion molecule 1, C-reactive protein, and angiopoetin 2 were measured by enzyme-linked immunosorbent assay at admission and at 7 day and 28 day follow-up visits in 84 children with uncomplicated malaria, 88 children with nonmalarial febrile illness, and 18 children with cerebral malaria. Results are compared with 36 Malawian healthy controls who were well children at the hospital for elective surgical procedures. Horizontal lines indicate geometric means, and bars indicate 95% confidence intervals. Numbers below data labels of the x-axis are the number of children in each group at each time point. Comparison was performed with a 1-way analysis of variance with the Tukey honestly significant difference test to adjust for multiple comparisons. Asterisks (*) indicate a statistically significant difference in comparison with the healthy controls: *P ≤ .05; **P ≤ .01 ***P ≤ .001. Abbreviations: Ang2, angiopoetin 2; CM, cerebral malaria; CRP, C-reactive protein; HC, healthy control; MF, nonmalarial febrile illness; sICAM-1, soluble intercellular adhesion moleculre 1; UM, uncomplicated malaria.

At enrollment (day 0), sICAM-1, Ang2, CRP, and sE-selectin concentrations were significantly higher in all febrile groups when compared with the HC group; sTM was raised in UM and CM patients, and F1 + 2 was raised in CM patients (Table 1, Figure 1, and Supplementary Figure 2). At 7 days postenrollment, CRP and Ang2 remained significantly raised in all groups when compared with the HC group. ICAM-1 remained raised in UM and CM patients and E-selectin, and F1 + 2 remained raised in CM patients only. At 28 days postenrollment, ICAM-1 and Ang2 remained significantly raised in MF and UM patients. CRP was significantly raised in CM patients only.

## DISCUSSION

The endothelial lining of blood vessels has a critical role in the regulation of blood flow, permeability, coagulation, inflammation, and innate and adaptive immunity [8]. A key determinant of endothelial function is the repertoire of surface receptors expressed, and these phenotypes are highly specific for the vascular beds of different organs [8]. Disruption of this surface receptor phenotype is critical in the pathogenesis of many diseases, including bacterial sepsis, cardiovascular disease, inflammatory bowel disease, and malaria [9, 10]. In acute *P. falciparum* malaria, cytokine- and parasite-mediated alteration of endothelial phenotype leads to endothelial dysfunction and a procoagulant state [10, 11]. In turn, the upregulation of several inducible endothelial surface receptors, including E-selectin and ICAM-1, facilitates the cytoadherence of *P. falciparum*–infected red blood cells (iRBCs) [10], a key component of the pathogenesis of CM [12]. It has been assumed that after each infection following iRBC elimination these perturbations resolve quickly and completely, with the endothelium rapidly returning to its predisease state. Here we show that endothelial activation and systemic inflammation persist after iRBCs are cleared from the circulation. This was most pronounced in CM, where CRP remained raised 22-fold higher at 28-day follow-up when compared with the HC group. However, significant inflammation and endothelial activation was also detectible in UM, with CRP remaining 13-fold above that of the HC group at 7-day follow-up and ICAM-1 and Ang2 remaining raised until the 28-day follow-up. This persistence cannot be explained by the half-lives of these factors, because, for example, CRP and Ang2 have half-lives of 19 hours [13] and 18 hours [14], respectively.

Persistent endothelial activation was not specific to malaria. In agreement with prior studies indicating persistent endothelial activation after other common infections [15], there was also endothelial activation in the group with mild nonmalarial infections. Nonetheless, the long-term effect of malaria might be anticipated to be particularly important in hyperendemic countries such as Malawi, where the frequency of repeated malaria infections may be sufficient to prevent return to baseline before the next infection (malarial or otherwise). Thus children who live in areas of high malaria transmission may be in a constantly deregulated endothelial state. This may influence outcome for patients in several different ways. First, subacute impairment of endothelial barrier function, as indicated here by increased Ang2, may increase invasion of bacteria or viruses into the blood stream. This mechanism has been proposed to explain the causal association between incidence of malaria and bacteremia shown using epidemiological modeling in Kenya [5]. Second, because endothelial activation is implicated in the pathogenesis of a number of infections, including malaria and bacterial sepsis, acquiring a subsequent infection while the endothelium remains activated may have an endothelial priming effect, increasing the vascular dysfunction and thus the severity of disease. Specifically, because parasites that bind ICAM-1 have been shown to be associated with CM [12], the residual ICAM-1 upregulation, indicated by raised sICAM-1 here, may select for parasite variants associated with higher incidence of CM. Finally, in the long term, chronic endothelial activation and inflammation may contribute to the endothelial changes that cause cardiovascular disease [15, 16]. In industrialized countries, the number of infectious episodes of common infections a person has accrued over their lifetime—their “pathogen burden”—has been implicated in risk of myocardial infarction in a dose–response fashion and is a better predictor of cardiovascular risk than any particular pathogen [15]. This indicates that although each individual infection may only cause a small residual insult to the vasculature, over multiple infectious episodes this may accumulate to cause severe pathology. Given the very high frequency of new malaria infections in many parts of sub–Saharan Africa, malaria may contribute substantially to this pathogen burden—even if the effect from each infectious episode is small. Although there is a paucity of epidemiological studies investigating the etiology of cardiovascular diseases in sub–Saharan Africa, these diseases are a significant cause of morbidity and mortality [17]. As a higher proportion of the population survives into and past middle age, their prevalence rates are likely to increase. Large epidemiological studies to identify the specific risk factors in Africans are needed, and our data indicate that malaria should be considered within these future studies.

There are several limitations to this study. First, loss to follow-up of 50% of the patients in each of the groups may have led to bias. Children with UM who attended follow-up had a significantly higher CRP on admission than those who did not attend follow-up (followed-up: mean, 137 mg/mL, 95% confidence interval [CI], 107–166 mg/mL; not followed-up, mean = 71 mg/mL, 95% CI, 50–93 mg/mL; *P* ≤ .01), which might indicate that sicker patients were more likely to return to follow-up, leading to an overestimation of the strength of difference with the HC group. However, comparison of all clinical variables and all biomarkers among all patients who were followed-up until 28 days with those who were lost to follow-up or excluded did not indicate a systematic bias (data not shown). Second, although the panel of plasma markers used are well validated as indicators of endothelial activation [9], only Ang2 and E-selectin are exclusively produced by the endothelium. In Indonesian adults with malaria, high Ang2 levels predicted impaired endothelial function, measured by endothelial-dependent vasodilatation [18]. In that population, these arterial tonometric measurements improved to within normal range by 14 days. It would be interesting to evaluate tonometry in African children to examine whether the raised Ang2 levels at 28 days here are associated with a persistently abnormal vasodilation response. Third, because it was not possible to measure preinfection levels of the biomarkers and we were not able to follow-up this cohort long-term, we have not proven that malaria causes the higher levels of biomarkers described at 28 day follow-up. It is possible, although we think unlikely, that instead patients in the febrile groups had preexisting higher baseline biomarker levels. Finally, because we studied children with symptomatic disease it remains unclear whether there is endothelial activation induced by parasitemia that is not associated with febrile illness, which would have important implications given the frequency of asymptomatic malaria infections.

Further research is needed to clarify the extent and duration of the endothelial perturbations described here and their relevance in other malaria-prevalent countrie and to ascertain whether burden of malaria infection is independently associated with adverse outcomes due to endothelial dysfunction in the short or long term. If such studies confirm a chronic effect of repeated vascular insults by malaria infection, that would have important implications: influencing the priority of preventing malaria and indicating a need to target interventions to older children and adults in addition to the young children who die from acute illness. A further consideration is the use of adjunctive therapies to prevent residual endothelial effects in individuals with severe malaria or a high burden of infection. A candidate is statins, which have endothelial protective, anti-inflammatory, and anticoagulant effects and prevent cardiovascular disease even in those without hyperlipidemia [16]. In malaria, statins reduce endothelial activation in vitro and reduce neurodevelopmental sequelae after experimental CM in mice [19]. Short courses of statins during acute illness and convalescence could be targeted to individuals with severe disease.

In conclusion, we demonstrate that in uncomplicated and severe malaria significant endothelial activation and inflammation persists for at least 1 month after elimination of iRBCs. Given the huge burden of repeated infection with malaria, this endothelial activation may represent a significant and previously neglected contribution to long-term health that warrants further evaluation for treatment and prevention strategies.

## Supplementary Data

Supplementary materials are available at *The Journal of Infectious Diseases* online (http://jid.oxfordjournals.org/). Supplementary materials consist of data provided by the author that are published to benefit the reader. The posted materials are not copyedited. The contents of all supplementary data are the sole responsibility of the authors. Questions or messages regarding errors should be addressed to the author.

Supplementary Data
