# Supplementary material for: Persistent Endothelial Activation and Inflammation After Plasmodium falciparum Infection in Malawian Children
Source: J Infect Dis. 2013 Sep 17;209(4):610–5. doi: 10.1093/infdis/jit419 (PMC3903368; doi:10.1093/infdis/jit419)
Supplement: Supplementary Data [file supp_209_4_610__index.html]

Persistent Endothelial Activation and Inflammation After Plasmodium falciparum Infection in Malawian Children — Persistent Endothelial Activation and Inflammation After Plasmodium falciparum Infection in Malawian Children — Supplementary Data 

# Persistent Endothelial Activation and Inflammation After *Plasmodium falciparum* Infection in Malawian Children

## Supplementary Data

Supplementary Data

**Files in this Data Supplement:**

- Supplementary Figure 1 - pptx file
- Supplementary Figure 2 - eps file
