# Supplementary material for: Persistent Endothelial Activation and Inflammation After Plasmodium falciparum Infection in Malawian Children
Source: J Infect Dis. 2013 Sep 17;209(4):610–5. doi: 10.1093/infdis/jit419 (PMC3903368; doi:10.1093/infdis/jit419)
Supplement: Supplementary Data [file supp_jit419_jit419supp_fig1.pptx]

## Slide 1
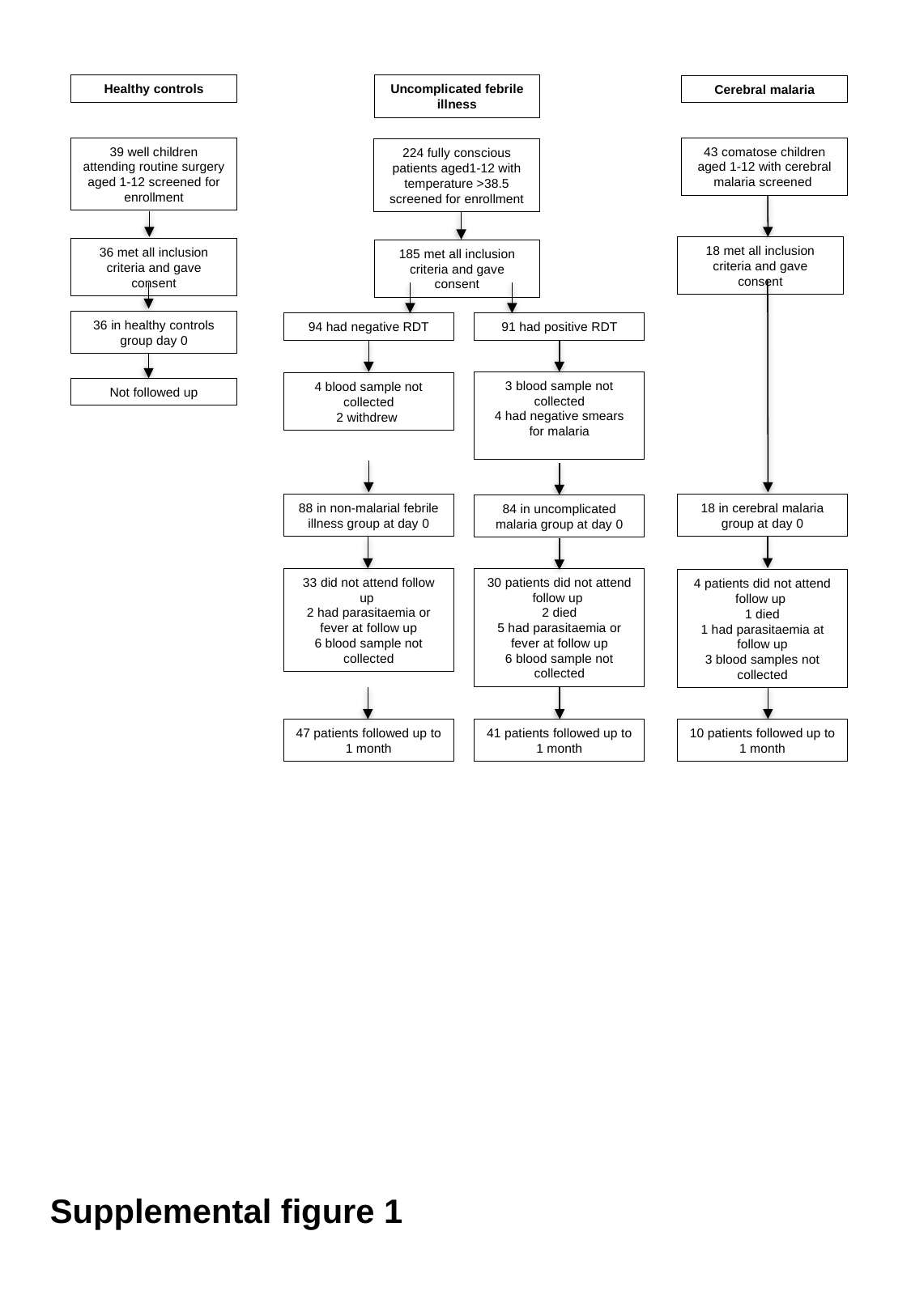

Healthy controls
Uncomplicated febrile illness
Cerebral malaria
39 well children attending routine surgery aged 1-12 screened for enrollment
43 comatose children aged 1-12 with cerebral malaria screened
224 fully conscious patients aged1-12 with temperature >38.5 screened for enrollment
18 met all inclusion criteria and gave consent
36 met all inclusion criteria and gave consent
185 met all inclusion criteria and gave consent
36 in healthy controls group day 0
94 had negative RDT
91 had positive RDT
3 blood sample not collected
4 had negative smears for malaria
4 blood sample not collected
2 withdrew
Not followed up
88 in non-malarial febrile illness group at day 0
18 in cerebral malaria group at day 0
84 in uncomplicated malaria group at day 0
33 did not attend follow up
2 had parasitaemia or fever at follow up
6 blood sample not collected
30 patients did not attend follow up
2 died
5 had parasitaemia or fever at follow up
6 blood sample not collected
4 patients did not attend follow up
1 died
1 had parasitaemia at follow up
3 blood samples not collected
10 patients followed up to 1 month
47 patients followed up to 1 month
41 patients followed up to 1 month
Supplemental figure 1
